# Genomic Alterations in Breast Cancer Patients in Betel Quid and Non Betel Quid Chewers

**DOI:** 10.1371/journal.pone.0043789

**Published:** 2012-08-24

**Authors:** Mishi Kaushal, Ashwani K. Mishra, Jagannath Sharma, Eric Zomawia, Amal Kataki, Sujala Kapur, Sunita Saxena

**Affiliations:** 1 National Institute of Pathology, ICMR, Safdarjung Hospital Campus, New Delhi, India; 2 Dr. B. Borrooah Cancer Institute, Guwahati, India; 3 Civil Hospital, Aizwal, India; Institut Jacques Monod, France

## Abstract

Betel Quid (BQ) chewing independently contributes to oral, hepatic and esophageal carcinomas. Strong association of breast cancer risk with BQ chewing in Northeast Indian population has been reported where this habit is prodigal. We investigated genomic alterations in breast cancer patients with and without BQ chewing exposure. Twenty six BQ chewers (BQC) and 17 non BQ chewer (NBQC) breast cancer patients from Northeast India were analyzed for genomic alterations and pathway networks using SNP array and IPA. BQC tumors showed significantly (P<0.01) higher total number of alterations, as compared with NBQC tumors, 48±17% versus 32±25 respectively. Incidence of gain in fragile sites in BQC tumors were significantly (P<0.001) higher as compared with NBQC tumors, 34 versus 23% respectively. Two chromosomal regions (7q33 and 21q22.13) were significantly (p<0.05) associated with BQC tumors while two regions (19p13.3–19p12 and 20q11.22) were significantly associated with NBQC tumors. GO terms oxidoreductase and aldo-keto reductase activity in BQC tumors in contrast to G-protein coupled receptor protein signaling pathway and cell surface receptor linked signal transduction in NBQC tumors were enriched in DAVID. One network “Drug Metabolism, Molecular Transport, Nucleic Acid Metabolism” including genes AKR1B1, AKR1B10, ETS2 etc in BQC and two networks “Molecular Transport, Nucleic Acid Metabolism, Small Molecule Biochemistry” and “Cellular Development, Embryonic Development, Organismal Development” including genes RPN2, EMR3, VAV1, NNAT and MUC16 etc were seen in NBQC. Common alterations (>30%) were seen in 27 regions. Three networks were significant in common regions with key roles of PTK2, RPN2, EMR3, VAV1, NNAT, MUC16, MYC and YWHAZ genes. These data show that breast cancer arising by environmental carcinogens exemplifies genetic alterations differing from those observed in the non exposed ones. A number of genetic changes are shared in both tumor groups considered as crucial in breast cancer progression.

## Introduction

Breast cancer is the most common malignancy worldwide among women attributed to various genetic and environmental factors [1]. In India it constitutes 22.2% of all cancers with approximately 115,000 incident cases reported in 2008 [2]. The several fold difference in incidence rates between different geographical regions suggest that environmental factors influence breast cancer risk significantly [3]. Both high and low age-adjusted breast cancer incidence rates (AAR) have been observed in Northeast India (23.3 in Aizwal to 12.1 in Dibrugarh in 2008) which has steadily increased [4].

A previous case control study on assessment of various environmental and genetic factors in Northeast Indian population illustrated significant increase in breast cancer risk in women who consumed Betel Quid (BQ) [3]. In the Northeast region of India BQ is consumed as a mixture of areca nut (Areca catechu), catechu (Acacia catechu) and slaked lime (calcium oxide and calcium hydroxide) wrapped in betel leaf (Piper betel) and tobacco [5].

BQ independently contributes to the risk of oropharyngeal cancer, oral mucosal lesions, oral leukoplakia, oral submucous fibrosis, liver cirrhosis and hepatocellular carcinoma [6]. In vitro and in vivo experiments have shown that BQ consumption can also cause micronuclei and DNA adducts formation, chromosomal aberrations, allelic imbalances and sister chromatid exchange in oral mucosa cells [7]. Carcinogens in BQ lead to accumulation of genetic alterations at 3q26.3 locus particularly in recurrent oral tumors [8] besides accelerating tumor migration by stimulating MMP-8 expression through MEK pathway [9].

In addition, calcium hydroxide a major content of slaked lime in the presence of areca nut is responsible for the formation of ROS (reactive oxygen species) known to cause oxidative damage in the DNA of buccal mucosa cells of BQ chewers. Presence of iron and copper transition metals are also involved in the catalytic process of ROS generation [5]. This ROS generation leads to structural alterations in DNA, including rearrangements, deletions, insertions and sequence amplification, affect cytoplasmic and nuclear signal transduction pathways, modulate the activity of the proteins and genes that respond to stress and act to regulate genes related to cell proliferation, differentiation and apoptosis [10].

Tobacco chewing with BQ results in increased exposure (∼1000 µg/day) to carcinogenic tobacco-specifc nitrosamines (TSNAs). High levels of TSNAs have been found in saliva samples of BQ chewers collected from India. N’-nitrosonornicotine (NNN), 4-(N-methyl-N-nitrosamino)-1-(3-pyridyl)-1-butanone (NNK), N-nitrosoanabasine (NAB), N-nitrosodimethylamine and N-nitrosodiethylamine have been detected in saliva of BQ with tobacco chewers [5], breast tissue of women workers and are known to induce mammary tumors in rodents and anaphase bridges via DNA double stranded breaks causing genomic imbalances in human cells [11], [12]. Regions like 7p11.2 (epidermal growth factor receptor) and 11q13.3 (cyclin D1) playing a role in pathogenesis of tobacco-related human squamous cell carcinoma has been identified by SNP array [13]. Examination of genomic alteration due to tobacco carcinogens depicts gain on chromosomes 6 and 8, and losses on chromosomes 11 and 14 in mouse lung adenocarcinomas [14] and gains of 1p and 3q in patients with tobacco exposure history in head and neck squamous cell carcinomas [15]. In addition, Benzo(*a*)pyrene [B(a)P] diolepoxide (BPDE), a carcinogen present in cigarette smoke, induces chromosomal 9p21 aberrations seen to be significantly higher in peripheral blood lymphocytes of bladder cancer cases than that of controls [16]. Allelic imbalance at 5q22.2∼q22.3 (LOX gene) is significantly higher among smokers than nonsmokers in clear cell renal carcinomas indicating that tobacco may cause genetic alterations [17].

Earlier studies on genomic alterations in breast cancer have investigated copy number changes between different subtypes and BRCA predisposed breast tumors and cell lines [18 19 20]. Although, the literature suggests role of BQ carcinogens in mediating genomic alterations, there is dearth of evidence suggesting its role in breast carcinogenesis. The present study has been undertaken to elucidate the genetic alterations induced by BQ chewing leading to breast carcinogenesis utilizing whole genome SNP array and Ingenuity pathway analysis in breast cancer patients with and without BQ chewing history.

## Results

### 10K SNP Array Profiles of Overall Breast Cancer Patients

Forty-three tissue samples of breast carcinoma and fourteen matched samples of germline DNA were analyzed for copy number alterations. The mean age of cases was 44.4±9.6 years and maximum cases were between 40–49 years. Twenty six patients were with BQ chewing history (BQC) and seventeen patients were without BQ chewing history (NBQC). Among the total cases, 23 cases were premenopausal and 20 cases were postmenopausal. Stage IV tumors were more, followed by stage III and II tumors whereas stage for six tumors was unknown. The association between above groups of patients with regard to patient age at diagnosis, tumor stage and menopausal status was statistically insignificant and no sample had a previous family history of cancer, alcohol drinking and tobacco smoking (Table S1). 110 recurrent altered regions were identified ranging from 0.15 Mb to 51 Mb in size (Table S2) with more gains than losses. More than 40% alterations were observed in 30 regions which were essentially gains (1q24.1, 1q25.2, 1q31.1, 1q32.1,1q41, 1q42.2–1q42.3, 1q43–44, 2p11.2, 5p13.3, 5p15.2, 7p12.3–7p12.1, 7p14.1–7p12.3, 7p14.3, 7p21.2, 7p21.3–7p21.2, 7q33, 8q12.1–8q12.2, 8q13.2, 8q22.1, 8q22.2, 8q22.3, 8q24.11, 8q24.21, 12q22.3, 16p13.3–16p13.2, 17q23.2, 17q23.3, 20q13.2, 20q13.33, 21q22.1. Most of the recurrent alterations observed were focal amplification (<10 Mb). Although, most chromosomes depicted multiple regions of alteration, 10, 22 and X chromosomes were altered in only single region with no alteration in chromosome 18. Frequent gains were observed in regions of long arm of chromosome 1 and 8 with genes implicated in cancer. 67 and 50 percent samples presented gain at 8q22.1 and 8q24 respectively. 1q43–44 and 1q41 regions presented with gain in 60 and 51 percent samples respectively. The remaining 80 regions were seen to be altered in 39 to 16 percent samples. The key regions comprising tumor associated genes were 6p25.3, 14q21.3, 1q21.1, 15q25.1, 1p13.2,20q13.11, 15q22.2–15q23, 19q13.11, 9p23,11q13.3, 11p14.3, 17q25.1, 3p24.2–3p23, 9q34.11–9q34.3, 5p15.33–5p12, 5q35.1–5q35.3, 22q12.1. Loss in single regions was more frequent than recurrent loss (losses in ≥15% of samples) (Table S2 and S3).

### Genetic Alterations Different between BQC and NBQC

**Figure 1:**
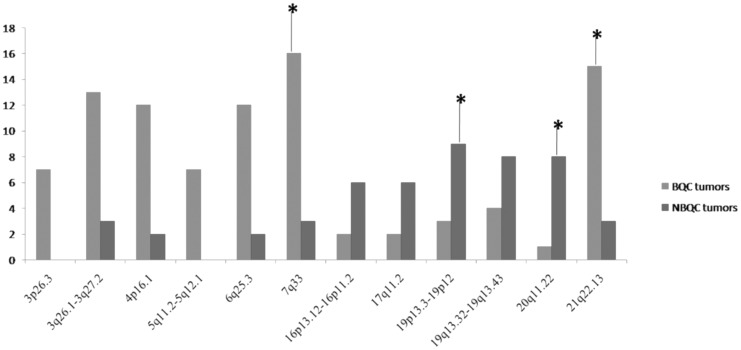
Chromosomal regions altered differently between BQC and NBQC breast tumors. *Regions significant after FDR correction.

Total number of alterations varied considerably between BQC and NBQC tumors from 12 to 93 alterations among BQC tumors, and from 6 to 63 alterations among NBQC tumors. The frequency plot of alterations per chromosome 1–X is shown in Figure S1. The BQC tumors showed a significantly (P<0.01, T test) higher total number of alterations, as compared with NBQC tumors (48±17% versus 32±25, respectively) (Table S3). One of the important finding was significantly high incidence of gain in fragile sites in BQC tumors (P<0.001, T test) as compared in NBQC tumors, 34 versus 23%, respectively (Table S2). Significant (P<0.05) differential genetic alterations were found in twelve chromosomal regions among BQC and NBQC tumors (Table 1, Figure 1). Among the twelve regions seven chromosomal regions (3p26.3, 3q26.1–3q27.2, 4p16.1, 5q11.2–5q12.1, 6q25.3, 7q33 and 21q22.13) presented more gain in BQC tumors while five regions (16p13.12–16p11.2, 17q11.2, 19p13.3–19p12, 19q13.32–19q13.43, 20q11.22) showed more gain in NBQC tumors. The alterations observed were chiefly gains of sizes ranging between 0.65 Mb to 22 Mb. Multiple testing was controlled using the false discovery rate (FDR) q-value method. The FDR cutoff up to 0.2 has been commonly used in case-control GWAS studies [21], [22]. FDR correction is likely to be conservative considering the relatively small number of cases, but four differentially altered regions at various chromosomes remained significant, as indicated by relatively low FDR values. The FDR value of 0.26 as for regions (3p26.3, 3q26.1–3q27.2, 4p16.1, 5q11.2–5q12.1, 6q25.3, 16p13.12–16p11.2, 17q11.2, 19q13.32–19q13.43) indicates that the relevance of these finding should be interpreted with caution, and we therefore focused particularly on the regions with P-values  = 0.01 and low FDR values. More than 50% BQC tumors presented with gain at 7q33 and 21q22.13 in contrast to just 17% gain in NBQC tumors. Among the regions altered more in NBQC tumors, 52% NBQC tumors had gain at 19p13.3–19p12 in comparison to gain in 11% BQC tumors and 47% of NBQC tumors had gain at 20q11.22 in comparison to gain in 3% BQC tumors.

**Table 1:** Chromosomal areas with gain those are significantly different between betel quid chewers (BQC) and non betel quid chewers (NBQC) breast cancer patients.

Cytoband	BQC (26)	NBQC (17)	P value	Q value (FDR)	Start Site	End Site	Size (Mb)
3p26.3	7	0	0.03	0.26	653347	2264798	1.61
3q26.1–3q27.2	13	3	0.05	0.26	165409849	167801377	2.39
4p16.1	12	2	0.02	0.26	10760950	11857265	1.09
5q11.2–5q12.1	7	0	0.03	0.26	57466589	58659721	1.19
6q25.3	12	2	0.02	0.26	155713132	157738990	2.02
**7q33**	**16**	**3**	**0.005**	**0.10**	**133281372**	**135010987**	**1.72**
**21q22.13**	**15**	**3**	**0.01**	**0.10**	**37974454**	**40484883**	**2.51**
16p13.12–16p11.2	2	6	0.04	0.26	10529386	33498455	22.96
17q11.2	2	6	0.04	0.26	22436842	23092917	0.65
**19p13.3–19p12**	**3**	**9**	**0.005**	**0.10**	**3542590**	**17471210**	**13.92**
19q13.32–19q13.43	4	8	0.03	0.26	51160543	63437743	12.27
**20q11.22**	**1**	**8**	**0.001**	**0.08**	**31982015**	**35933409**	**3.95**

Frequency of chromosomal regions with significantly different (P<0.05; see Materials and methods for the statistical test) alterations between TBC and NTBC tumors are depicted. Most significant regions, based on the criteria of P<0.05 and a relatively low FDR value, are indicated in bold. FDR  =  false discovery rate.

### Gene Ontology (GO) and Network Analyses of Associated Regions

**Figure 2:**
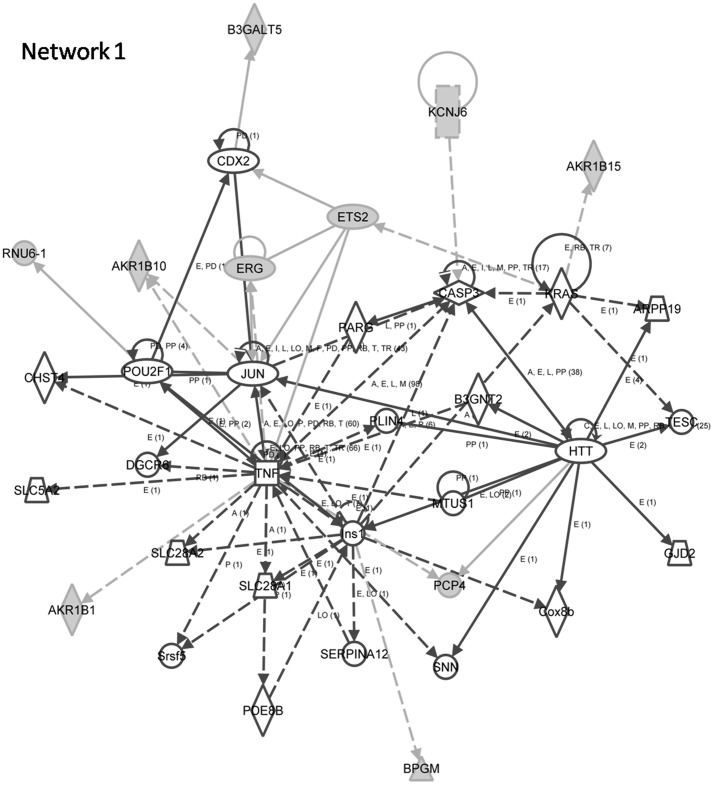
BQC Network 1 Drug Metabolism, Molecular Transport, Nucleic Acid Metabolism.

**Figure 3:**
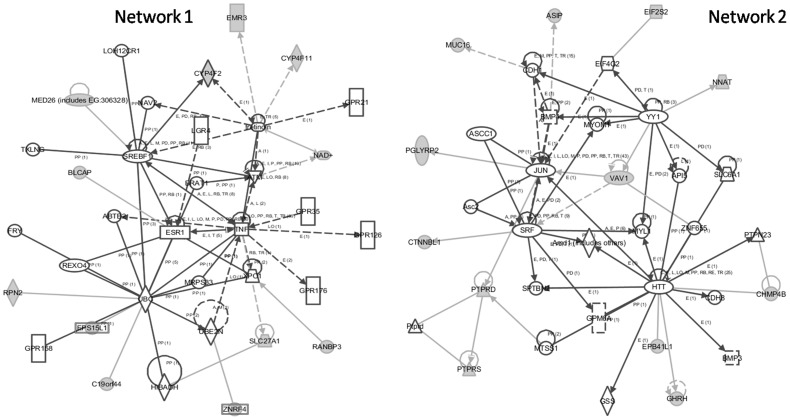
NBQC Networks: Molecular Transport, Nucleic Acid Metabolism, Small Molecule Biochemistry (Network 1) and Cellular Development, Embryonic Development, Organismal Development (Network 2).

Genes associated with BQC regions, 7q33 and 21q22.1 were enriched for oxidoreductase (p<0.001) and aldo-keto reductase activity (p = 0.015) in contrast to G-protein coupled receptor protein signaling pathway (p = 0.005) and cell surface receptor linked signal transduction (p = 0.012) for 19p13.3–19p12 and 20q11.22 NBQC associated regions. IPA (Ingenuity Pathway Analysis) analysis for BQC associated regions revealed one top network (score = 20) “Drug Metabolism, Molecular Transport, Nucleic Acid Metabolism” encompassing genes like AKR1B1, AKR1B10, AKR1B15, ERG, ETS2 (Figure 2). IPA analysis for NBQC genes revealed two top networks (score = 29) “Molecular Transport, Nucleic Acid Metabolism, Small Molecule Biochemistry” and “Cellular Development, Embryonic Development, Organismal Development” (Figure 3) encompassing genes like RPN2, EMR3, BLCAP and VAV1, NNAT and MUC16 respectively.

### Genetic Alterations Similar between BQC and NBQC

Twenty seven common regions of gain were illustrated between BQC and NBQC tumors. Regions demonstrating gain in minimum 30% cases from each group were considered as similarly altered [23]. Both groups exhibited gain on chromosomes 1q, 5p, 7p, 8q, 12q, 16p, 17q and 20q (Table S4). Gain in more than 50% samples was seen in six regions (1q31.1, 1q42.2–1q42.3, 1q43–44, 8q22.1, 8q22.2, 8q24.11). Gain in more than 45% samples was seen at 1q24.1, 1q41, 7p12.3–7p12.1, 8q24.21 and 20q13.2 regions. Other regions encompassing probable tumor associated genes were 1q32.1, 1q21.1, 7p21.3–7p21.2, 12q22.3, 16p13.3–16p13.2, 17q23.3.

### Gene Ontology (GO) and Network Analyses of Similar Regions

**Figure 4:**
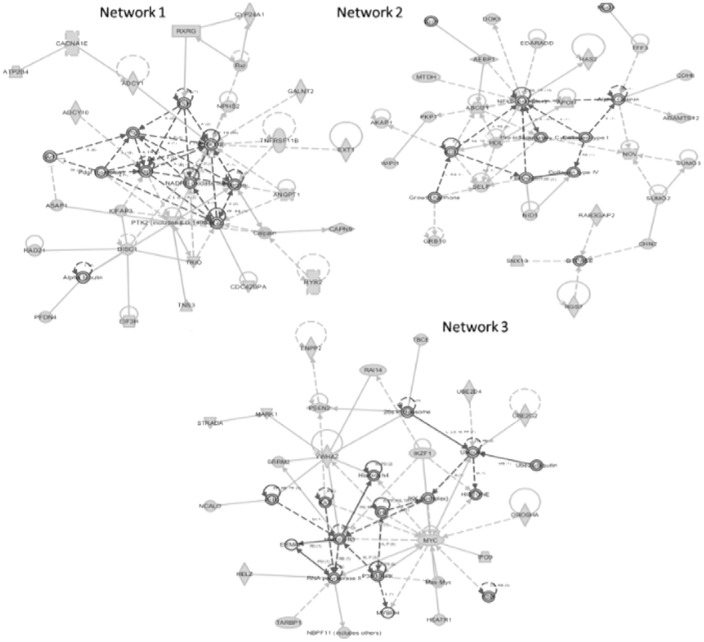
Common Networks: Cellular Movement, Connective Tissue Development and Function, Cellular Assembly and Organization (Network 1), Cell-To-Cell Signaling and Interaction, Tissue Development, Organismal Injury and Abnormalities (Network 2), Cell Morphology, Cellular Assembly and Organization, Cellular Compromise (Network 3).

**Figure 5:**
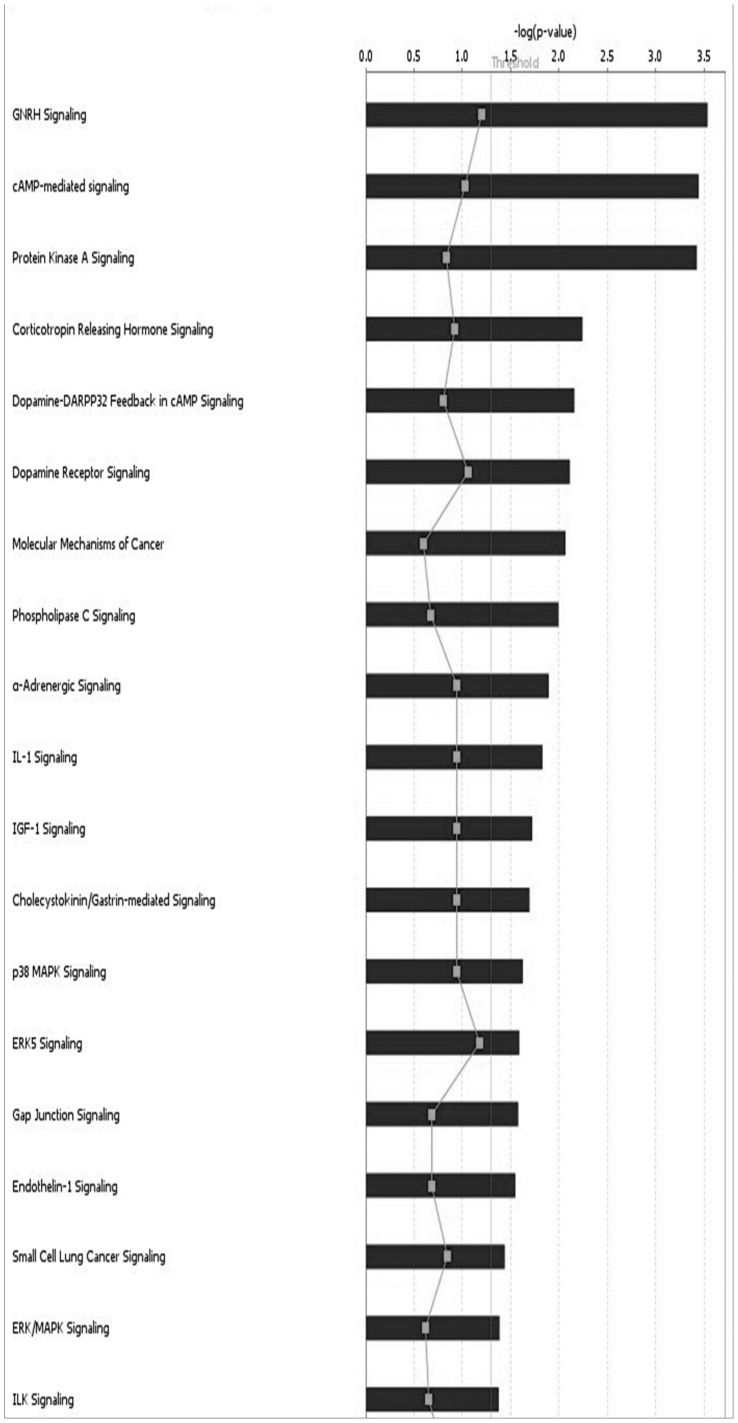
Cancer related Canonical pathways enriched from common genes.

Enrichment and IPA was performed to investigate the function of genes associated with these regions. Regions were mainly enriched for activation of protein kinase activity (p = 0.009), cell junction (p = 0.01). IPA analysis revealed three top networks (Table 2, (Figure 4). Network 1 functions in Cellular Movement, Connective Tissue Development and Function, Cellular Assembly and Organization (score = 43) with key role played by PTK2. Network 2 functions in Cell-To-Cell Signaling and Interaction, Tissue Development, Organismal Injury and Abnormalities (score = 43) with RPN2, EMR3, VAV1, NNAT and MUC16 important genes. Network 3 functions in Cell Morphology, Cellular Assembly and Organization, Cellular Compromise (score = 32) with key roles played by MYC and YWHAZ. Among all the tumor associated canonical pathways enriched were GNRH signaling (p = 2.92E−04), cAMP-mediated signaling (p = 3.60E−04), Protein Kinase A signaling (p = 3.77E−04), CXCR4 signaling (p = 4.99E−03), molecular mechanisms of cancer (p = 8.58E−03), phospholipase C Signaling (p = 1.01E−02), RAR Activation (p = 3.16E−02), ILK Signaling (p = 4.21E−02)(Table S5, Figure 5).

**Table 2:** Significant signaling pathway networks observed in BQC, NBQC and Common altered genomic regions.

Networks	Nodes (genes) in Network	Score	Nodes	Identified Nodes	Top Functions
BQC Network 1	AKR1B1,AKR1B10,AKR1B15,ARPP19, B3GALT5,B3GNT2,BPGM,CASP3,CDX2,CHST4,Cox8b,DGCR6,ERG,ETS2,GJD2,HTT,Ins1,JUN, KCNJ6,KRAS,MTUS1,PARG,PCP4,PDE8B,PLIN4, POU2F1,RNU6-1,SERPINA12,SLC28A1,SLC28A2, SLC5A2,SNN,Srsf5,TESC,TNF	28	34	10	Drug Metabolism, Molecular Transport, Nucleic Acid Metabolism
NBQC Network 1	ABTB2,ATM,BLCAP,BRAT1,C19orf44, CYP4F2,CYP4F11,EMR3,EPS15L1, ESR1,FRY,GPR21,GPR35,GPR126, GPR158,GPR176,HIBADH,LGR4,LOH12CR1,MED26 (includes EG:306328), MRPS33,NAD+,NAV2,RANBP3,REXO4, RPN2,SLC27A1,SREBF1,TNF,tretinoin,TXLNG,UBC,UBE2N,XPO1,ZNRF4	29	34	12	Molecular Transport, Nucleic Acid Metabolism, Small Molecule Biochemistry
NBQC Network 2	Amd1 (includes others),API5,Asc2,ASCC1, ASIP,BMP3,BMP4,CDH1,CDH8,CHMP4B, CTNNBL1,EIF2S2,EIF4G2,EPB41L1,GHRH,GPM6A, GSS,HTT,JUN,MTSS1,MUC16,MYL1,MYOM1,NNAT, PGLYRP2,PTPN23,PTPRD,Ptprd,PTPRS,SLC6A1,SPTBN2,SRF,VAV1,YY1,ZNF655	29	34	12	Cellular Development, Embryonic Development, Organismal Development
COMMON Network 1	ADCY1,ADCY10,Alpha tubulin,ANGPT1,Arf, ASAP1,ATP2B4,CACNA1E,Calpain,CAPN9, CDC42BPA,CYP24A1,DISC1,EIF3H,ERK1/2, EXT1,GALNT2,Integrin,KIFAP3,NADPH oxidase, NPHS2,Pdgf (complex),PFDN4,Pld,PTK2 (includes EG:14083),Rac,RAD21,Rap1,Rxr, RXRG,RYR2,TNFRSF11B,TNS3,TRIO,TSH	43	35	25	Cellular Movement, Connective Tissue Development and Function, Cellular Assembly and Organization
COMMON Network 2	ABCG1,ADAMTS12,AEBP1,AKAP1, Alpha catenin,APOH,CDH6,CHN2, Collagen type I,Collagen type IV,DOK5, Ecm,EDARADD,Fibrinogen,GRB10, Growth hormone,GTPASE,HAS2,HDL,LDL, MTDH,Mucin,NFkB (complex),NID1,NOV, PKP1,Pro-inflammatory Cytokine,RAB3GAP2, RGS7,SELP,SNX13,SUMO2,SUMO3,TFF3,WIPI1	43	35	24	Cell-To-Cell Signaling and Interaction, Tissue Development, Organismal Injury and Abnormalities
COMMON Network 3	26s Proteasome,Actin,Ck2,DROSHA,ENPP2, ERMAP,FSH,HEATR1,HELZ,HISTONE, Histone h3,Histone h4,IKK (complex),IKZF1, IPO9,Jnk,MARK1,Max-Myc,MYBPH,MYC, NBPF11 (includes others),NCALD,P38 MAPK, PSEN2,RAI14,RNA polymerase II,SRRM2,STRADA, TARBP1,TBCE,Ube2-ubiquitin,UBE2D4,UBE2G2, Ubiquitin,YWHAZ	32	35	20	Cell Morphology, Cellular Assembly and Organization, Cellular Compromise

## Discussion

Two chromosomal regions, 7q33 and 21q22.3 presented more alterations in BQC tumors (gains) than NBQC tumors. Gain of 7q33 region has been previously reported in pancreatic and lung carcinoma [23], [24]. Gain of 21q22.3 has previously been described in cholangiocarcinoma and as one of the predictive marker regions of systemic recurrence in breast cancer [25], [26]. GO terms, oxidoreductase and aldo-keto reductase activity were enriched with a single drug metabolism, molecular transport, nucleic acid metabolism network. AKR1B1and AKR1B10 genes were seen playing cardinal roles. AKR1B10 is overexpressed in colorectal, uterine, breast cancers. Considered a diagnostic marker in lung cancer, it may play a pathogenic role in hepatocellular carcinoma. Role of AKR1B10 in tobacco-related carcinogenesis is anticipated because of its overexpression observed in bronchial epithelium of smokers. Its expression which is stimulated by tobacco smoke condensate in normal human epidermal, oral and squamous cell carcinoma cells decreases with the cessation of smoking. Proposed AKR1B10-mediated tumorigenic mechanisms include retinoic acid depletion and cancer cell dedifferentiation as well as chemoresistance due to metabolism of carbonyl group–bearing anticancer drugs and activating pro-carcinogens and polycyclic aromatic hydrocarbon (PAH) transdihydrodiols to biologically reactive and redox-active *o*quinones [27], [28], [29]. Hence, the tobacco component in BQ may explain AKR1B10 gain rendering chemoresistance, dedifferentiation and DNA adduct formation in BQC leading to breast carcinogenesis. In addition, AKR1B1 contributes in regulating multiple inflammatory pathways and its inhibition has been shown to interrupt inflammation triggered by chemokines, growth factors and inflammatory cytokines such as TNF-α as depicted in our network [29]. The ROS generated by the presence of slaked lime in BQ may amplify AKR1B1 gene rendering TNFα induced proliferation of breast cancer cells. Besides, the above network analysis also manifested role of ETS2 gene which maintains hTERT gene expression by interacting with the c-Myc transcription factor. It is a central driver of a transcriptional program in tumor associated macrophages that acts to promote lung metastasis of breast tumors [30], [31].

Our data also presented with a high significance of gain in fragile sites in BQC tumors as compared with NBQC. Fragile sites form gaps, constrictions and breaks on chromosomes when exposed to partial replication stress and are rearranged in tumors. Frequency of fragile sites and sister chromatid exchanges have been found to be significantly higher in smokers in peripheral lymphocytes and bone marrow [32], [33]. The above ascertains the potential of BQ carcinogens in causing chromosomal damage and instability leading to genetic alterations.

Since metabolic absorption of the ingredients of BQ directs the cancer-causing principles to other organs/tissues of the body, the evidence is growing to indicate that cancers other than oropharyngeal may also be caused by BQ chewing [34]. Tobacco related carcinogens can be stored in breast adipose tissue, metabolized and activated by human mammary epithelial cells [35]. Moreover, the evidence that tobacco exposure (smoking) causes early gene expression changes in normal airway epithelial cells and many other cancer types [36], the aforesaid observed changes likely reflect early carcinogenesis.

Among the NBQC tumors, two regions 19p13.3–19p12 and 20q11.22 presented more alterations. Gain observed in 19p13.3–19p12 has previously been reported in cutaneous and oral squamous cell carcinomas [37], [38]. Gain at 20q11.2 has been observed in breast, colorectal and cervical cancers [39], [40], [41]. GO terms, G-protein coupled receptor protein signaling pathway and cell surface receptor linked signal transduction were enriched with two networks, molecular transport, nucleic acid metabolism, small molecule biochemistry and cellular development, embryonic development, organismal development. RPN2, EMR3, VAV1, NNAT and MUC16 genes were recognized to have imperative functions. A recent study by Kimi Honma *et al* reported that RPN2 silencing and downregulation makes cancer cells hypersensitive in response to docetaxel a chemotherapeutic drug, proposing it as a target for RNA interference–based therapeutics against drug resistance [42]. EMR-3 a G-protein coupled receptor, upregulated in glioblastoma is associated with poor survival and is a potential mediator of cellular invasion [43]. VAV1 contributes to tumorgenesis by regulating both cellular proliferation and cell survival pathways through the regulation of an EGF-Src-Vav1-Rac1-Pak1-NF-κ B-Cyclin D1 signaling axis. An increased and ectopic expression of VAV1 in lung and pancreatic tumors has been linked to large tumors and worse survival rate respectively [44], [45]. mRNA expression of neuronatin (NNAT) has been reported in pituitary adenoma, prostatic cancer with neuroendocrine features, large cell neuroendocrine carcinoma lung and thyroid stimulating hormone-producing tumors in mice. High expression has been reported in a tamoxifen-resistant mammary carcinoma cell line [46]. Decreasing MUC16 levels are known to be of prognostic outcome in the post-operative and pre-operative neo-adjuvant chemotherapy especially in ovarian carcinoma [47]. Recent studies by Silke Reinartz *et al* and I Lakshmanan *et al* elucidated its central role in adhesion, migration and invasion in breast cancer. Overexpressed in breast cancer, it augments cell proliferation by interacting with JAK2 and inhibiting the apoptotic process through downregulation of TRAIL [48], [49]. Since the NBQC tumors had no previous history of BQ and any other environmental exposure, gain in genes regulating various facets of tumorigenesis can only be blamed as spontaneous instances arising in NBQC tumors.

Besides differences, both tumor groups shared twenty seven frequently altered regions. The IPA analyses resulted in three top networks and eight tumor associated canonical pathways. Extrapolating the data depicted ERK 1/2 and PTK2 (network 1), NFkB complex, SELP and NOV (network 2), MYC and YWHAZ (network 3) were key nodes in their respective networks.

A review by Ming Luo *et al* on PTK2 (FAK) described its principal role in breast carcinogenesis. As depicted in our network 1, PTK2 served as a mediator of cell cycle regulation by integrins through PTK2/Src complex formation in the focal contacts promoting ERK activation. Mechanistic studies indicate that PTK2 deletion in mammary tumor cells reduces the expression/phosphorylation of ERK1/2 contributing to the tumor dormancy in *vivo* and arrests growth in cultures suggesting PTK2 signaling through ERK-MAPK pathway is required to maintain tumor cell growth. In addition to Rac, PTK2 also mediates the activation of ERK to promote cell migration [50].

Network 2 witnessed SELP, NOV and NFκB complex as vital genes. NF-kB plays a key role in regulating the immune response and incorrect regulation of NF-kB has been linked to the development of cancer. Signaling pathways leading to tamoxifen resistance in breast cancer share a common mechanistic link with activation of nuclear factor-kB (NFkB) [51]. Elevated levels of SELP have been observed in many cancers including melanoma, tongue, colon, gastric, lung and breast. SELP is an adhesion molecules that mediate cell-cell interactions among platelets) and endothelial cells. Its measurement may provide a sensitive tool for monitoring the clinical course of melanoma and lymphoma [52]. High expression levels of NOV are associated with endocrine therapy crossresistance in CL6.7 cells and endocrine therapy resistance in breast tumor samples proliferation [53]. NOV enhances migration of chondrosarcoma cells by increasing MMP-13 expression through αvβ3/αvβ5 integrin receptor, FAK, PI3K, Akt, p65, and NF-κB signal transduction pathway and regulates the differentiation of bone resident cells creating a resorptive environment that promotes the formation of osteolytic breast cancer metastases [54], [55].

YWHAZ (14-3–3ζ) seen in network 3, overexpressed in breast, lung and many other cancers is implicated in the initiation and progression of cancer [56]. Low level copy number gains in YWHAZ have been found in head and neck squamous cell carcinomas [57]. Previous studies documenting YWHAZ upregulation and a poor clinical outcome in tamoxifen treated breast cancer patients imply it to be a marker of poor prognosis in women with ER-positive breast cancers [58]. The oncogenic Myc protein in network 3 plays an important role in breast cancer metastasis and several transcription factors are involved in the regulation of Myc expression. In breast cancer, amplification of c- *myc* may correlate positively or negatively with alterations in other genes [59]. For e.g. as revealed by our network 3 heterodimerization with Max is necessary for c-Myc to mediate proliferation, transformation, and apoptosis [60]. Recent studies have indicated that Myc is an IKKs substrate and IKKs tightly regulate Myc expression in breast cancers as also seen in network 3 [61].

Alterations seen in the preceding genes can be seen as vital as they arise independently of the etiological factors signifying the abovementioned genes importance in breast tumorigenesis. In addition, direct or indirect association of these key network genes to other cancer related genes (for example, MTDH, EXT1, ANGPT1, RAD21, EDARADD, TFF3, MARK1, DROSHA, etc seen in our networks) could create a permissive context activating or deactivating various facets of breast tumorigenesis. Super inducing these common alterations, AKR1B10, AKR1B1 and ETS2 alterations were BQ induced whereas alterations in RPN2, EMR3, VAV1, NNAT and MUC16 genes in NBQC tumors could only be termed as spontaneous.

It is important to acknowledge that apart from environmental factor such as betel quid being the prime focus of this study, genetic risk factors such as BRCA1 and BRCA2, lifestyle risk factors such as diet and reproductive risk factors also contribute to breast cancer. BRCA1 and BRCA2 mutant carriers impose a highly increased risk for hereditary or familial breast cancer. While our study is specifically based on sporadic tumors, BRCA2 mutation analysis performed on a larger set of samples in our unpublished study showed none of the tumors to be BRCA2 mutation positive. Therefore, likelihood of our samples containing BRCA1 mutations would still be minute if the probability of BRCA1 and BRCA2 mutations taken together is estimated to be 5%, equal to the proportion in total breast cancer incidence. Examination of impact of BRCA1 and BRCA2 mutations on copy number alteration illustrates a significant difference of genomic profiles between BRCA1 and sporadic tumors, followed by BRCA1 and BRCA2 tumors. BRCA2 and sporadic tumors (such as in our study) had very similar genomic profiles. Overall, BRCA1 tumors have a higher frequency of copy number alterations [62] implying that high risk cases of BRCA1 mutant carriers if subjected to environmental toxicants like betel quid could exemplify the effects resulting in aggressive and early tumors. Furthermore, lifestyle factor like diet has been implicated as an important determinant of breast cancer. The diet pattern in Northeast population of India is mainly characterized by high intakes of dry fish and fermented soybean and vegetables [63]. Such dietary pattern rich in vegetables and fish, but poor in red meats and animal fats has been positively associated to a longer overall survival of breast cancer. However obese women have increased risk for breast cancer as they are exposed to high levels of estrogen additionally produced by adipose tissue [64]. Reproductive factors, including age at menarche, age at first full-term pregnancy, number of live births and breast-feeding are related to a risk of breast cancer. Mechanism through which reproductive exposures influence breast cancer risk is their effect on lifetime number of menstrual cycles. Number of menstrual cycles influences the lifetime exposure to endogenous ovarian hormones like estrogen, which is strongly related to breast cancer risk [65]. Estrogen when metabolized produces metabolites which further contribute to tumor initiation by activating estrogen receptor and generating DNA damaging molecular species [66]. In our unpublished study breast cancer risk was not associated with any of the reproductive factors and polymorphism in an estrogen synthesizing CYP17 gene in the Northeast population of India (3). However, examination of the effect of lifestyle factors and reproductive factors on copy number alteration yet remains to be investigated. The foregoing further ascertains that the effects seen in the present study are due to betel quid chewing.

To our knowledge this is the first report of comparison of genomic alterations between BQ and NBQ chewer breast cancer patients. Overall our data agree well with previous genomic alteration analysis. The major strength of this study is its homogeneous sample population, presence of only BQ as an environmental exposure variables and detailed demographic information. As a limitation, analysis of a larger sample set and cell systems is clearly needed to more precisely delineate the molecular basis for both BQC and NBQC breast tumors. Despite that the accuracy of our results is justified due to unbiased sample distribution in both groups and FDR adjustments. Since composition of BQ in this region consists of multiple components, assessing carcinogenic effect of individual constituent was not possible in this study. Application of high resolution arrays may elicit additional regions of differential alteration. Unfortunately, such studies are largely precluded by the relative rarity of appropriate specimens. However, biological information obtained from BQ exposed breast cancer subset is valuable. This subgroup is frequent in the North East Indian population as most of the women in this area are usually chewers. Given a unique set of underlying genomic changes, distinct approaches to treatment may be appropriate for this patient population and others where this habit is highly prevalent.

## Materials and Methods

### Patient Recruitment and Sample Collection

Ninety two patients with breast tumors histopathologically confirmed as breast cancer at the Dr. B. Borrooah Cancer Institute, Guwahati and Civil Hospital, Aizwal India between November 2005 and December 2008 were registered for this study. Besides collecting tumor tissues in formalin for histopathology, tumor tissue in RNAlater and 5 ml blood in EDTA vials were collected for copy number analysis. Demographics, including age, sex, menopausal status, BQ history, tobacco history, alcohol drinking, family history and area of residence were obtained for each case. To quantify betel quid chewing we defined a habitual BQ chewer who chewed one betel quid or more daily for no less than ten years. Details of betel quid chewing history for 26 BQC samples are given in supplementary table S6. The ingredients of BQ included areca nut (Areca catechu), catechu (Acacia catechu) and slaked lime (calcium oxide and calcium hydroxide) wrapped in a betel leaf (Piper betle) and tobacco. Thirty two patients with locally advanced breast cancer were given neoadjuvant chemotherapy therefore were excluded. DNA was extracted from the fresh frozen tumor tissue and blood. Specimens with lower than 70% cancer cellularity, inadequate DNA concentration (<50 ng/mL), or a smearing pattern in gel electrophoresis were not included for genotyping. On this basis 43 cases of breast cancer cases were selected and analyzed for copy number assessment which included 26 BQC with only BQ chewing history and 17 NBQC with no history of tobacco chewing, tobacco smoking and alcohol consumption. All 43 cases were morphologically infilterating ductal carcinoma, not otherwise specific. Control germline DNA extracted from blood lymphocytes was used from age matched 14 breast cancer patients. All samples were collected with the patient’s written informed consent and the study was approved by the institutional ethics committee of Regional Medical Research Centre, North East Region (Indian Council of Medical Research).

### Single Nucleotide Polymorphism Array

Genechip Mapping 10 K early access array analysis The Single Primer Assay Protocol (labeling, hybridization, washing, staining and scanning) was performed according to the manufacturer’s instructions (Affymetrix, Santa Clara, CA, USA).

### Data Analysis

The primary experimental data was normalized to a baseline array with median signal intensity by applying invariant set normalization method. Copy number change was measured based on comparing the signal intensities at each probe locus between control and tumor samples by applying the hidden Markov Model using the dChip software, with a sliding window of 3 SNPs. Copy number gain was defined as >2.8 copies and loss was defined as less than 1.2 copies in at least 3 consecutive SNPs [67]. Recurrent altered regions were identified as regions with gain or loss in ≥3SNPs in not less than 15% of samples [68]. Mapping information of SNP locations and cytogenetic band were based on curation of Affymetrix and University of California Santa Cruz hg 17 (http://genome.ucsc.edu). To identify exposure-related aberrations, the data from individual patients were analyzed at group level by comparing gene copy number ratios of the tumors of chewer and nonchewer patients. In each region, we considered a 3×2 contingency table, with the rows representing number of patients with copy number gain, copy number loss or normal copy number in that region and the column representing BQC and NBQC breast cancer patients. Significant regions (p<0.05) were identified by comparing the copy number changes in the 26 BQC versus 17 NBQC breast cancer patients using a Fisher’s Exact Test based on the 3×2 table in each region. FDR was calculated using Benjamini and Hoeschbergs using the Q value software in R package [69].

### Gene Ontology (GO), Pathway and Network Analyses

Functional annotation analysis was performed using the DAVID (Database for Annotation, Visualization and Integrated Discovery) Functional Annotation Tool and Database [70]. A modified, more conservative Fisher’s exact p-value, or EASE score, is used to determine if there is a significant level of enrichment in the gene set. To determine pathways and networks those were significantly enriched in the two groups we performed pathway analysis using the Ingenuity Pathway Analysis (IPA) program (http://www.ingenuity.com).

## Supporting Information

Figure S1 **Prevalence (%) of patients with ≥3 copies (red) and ≤1 copies (blue) in BQC and NBQC tumors, respectively. The x-axis represents the positions in genome/chromosomes, and the y-axis represents the prevalence.**
(TIF)

Table S1 **Patient and tumor characteristics in relation to betel quid chewing.**
(DOC)

Table S2 **Total 110 regions seen to be altered in overall samples.**
(XLS)

Table S3 **Chromosomal gains and deletions in breast tumors from 26 BQC and 17 NBQC.** High-level amplifications are in boldface.
(XLS)

Table S4 **Regions with chromosomal alterations frequent in betel quid chewers and non chewers breast cancer patients.**
(XLS)

Table S5 **DAVID analysis of genes in BQC, NBQC and Common regions.**
(XLS)

Table S6 **Details of betel quid chewing history for 26 BQC samples.**
(DOC)
