# Supplementary figures and images for: Genomic Alterations in Breast Cancer Patients in Betel Quid and Non Betel Quid Chewers
Source: PLoS One. 2012 Aug 24;7(8):e43789. doi: 10.1371/journal.pone.0043789 (PMC3427153; doi:10.1371/journal.pone.0043789)

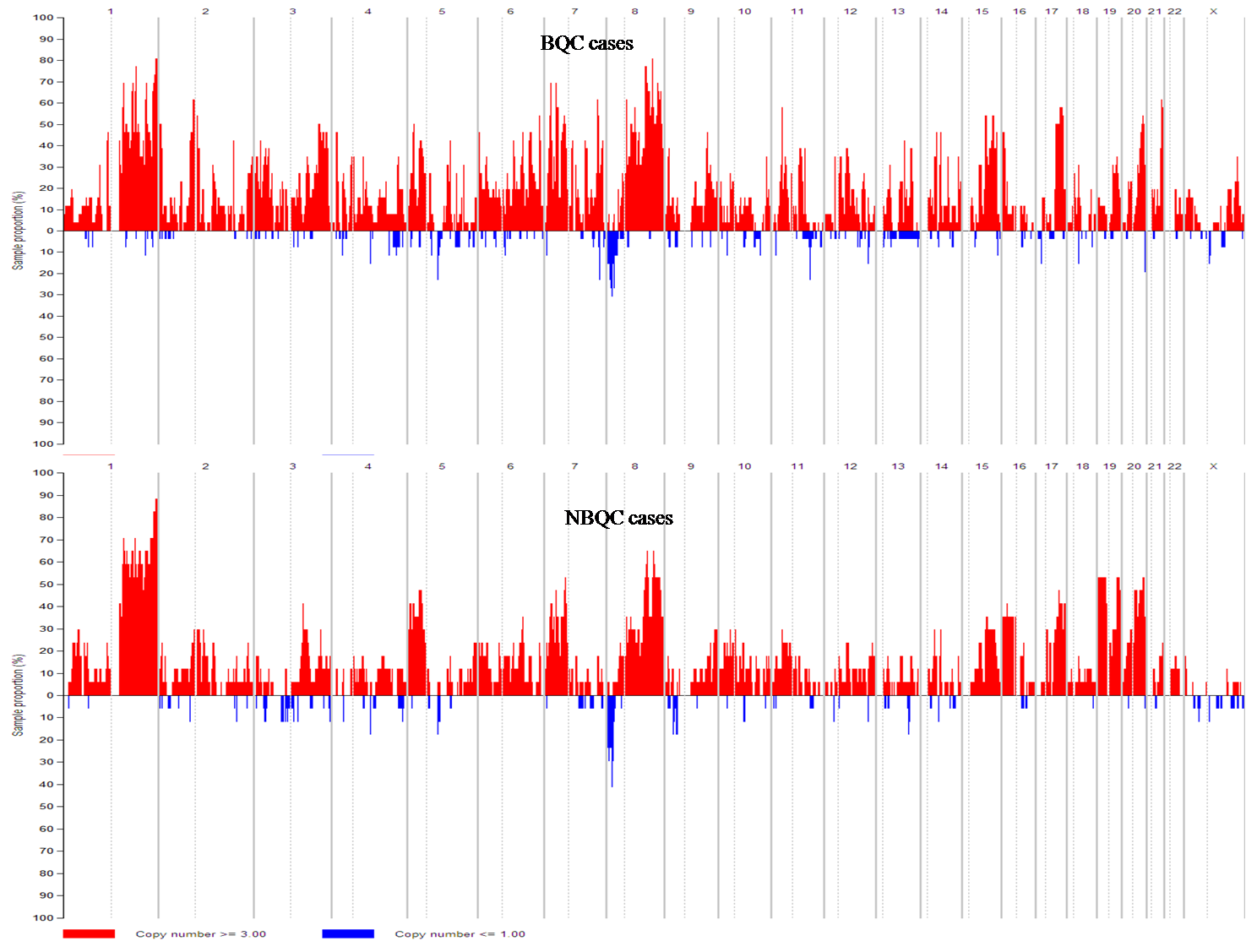

Supplement: Figure S1 — Prevalence (%) of patients with ≥3 copies (red) and ≤1 copies (blue) in BQC and NBQC tumors, respectively. The x-axis represents the positions in genome/chromosomes, and the y-axis represents the prevalence. (TIF) [file pone.0043789.s001.tif]
