# Supplementary material for: Genomic Alterations in Breast Cancer Patients in Betel Quid and Non Betel Quid Chewers
Source: PLoS One. 2012 Aug 24;7(8):e43789. doi: 10.1371/journal.pone.0043789 (PMC3427153; doi:10.1371/journal.pone.0043789)
Supplement: Table S1 — Patient and tumor characteristics in relation to betel quid chewing. (DOC) [file pone.0043789.s002.doc]

**Table S1: Patient and tumor characteristics in relation to betel quid chewing**

| **Variable** | **BQC 26 (%)** | **NBQC 17 (%)** | **P value** |
| --- | --- | --- | --- |
| Age at diagnosis |  |  |  |
| Mean | 44.63 ± 5.8 | 44.3 ± 11.6 | 0.9 |
| TMN Stage |  |  |  |
| II | 9 (34.6) | 4 (25.5) | 0.28 |
| III | 4 (15.3) | 2 (11.7) |  |
| IV | 8 (30.7) | 10 (58.8) |  |
| unknown | 5 (19.2) | 1 (5.8) |  |
| Menopausal status |  |  |  |
| Premenopausal | 15 (57.6) | 8 (47.0) | 0.54 |
| Postmenopausal | 11 (42.3) | 9 (52.9) |  |
| Family history |  |  |  |
| Yes | 0 | 0 |  |
| no | 26 | 17 |  |

Fisher’s exact test was used. A P-value <0.05 was considered to reflect a significant difference.
