# Supplementary material for: Genomic Alterations in Breast Cancer Patients in Betel Quid and Non Betel Quid Chewers
Source: PLoS One. 2012 Aug 24;7(8):e43789. doi: 10.1371/journal.pone.0043789 (PMC3427153; doi:10.1371/journal.pone.0043789)
Supplement: Table S6 — Details of betel quid chewing history for 26 BQC samples. (DOC) [file pone.0043789.s007.doc]

**Table S6: Details of betel quid chewing history for 26 BQC samples**

| Sno | Sample ID | Betel quid chewing (Yrs) | Number of Betel quid chewed per day. |
| --- | --- | --- | --- |
| 1 | 13T-371-06-T-GW | 40 | 11 |
| 2 | 14T-143-06-T-GW | 30 | 3 |
| 3 | 17T-372-06-T-GW_rs | 30 | 8 |
| 4 | 18T-530-08-T-GW_rs | 10 | 2 |
| 5 | 20T-531-08-T-GW_rs | 22 | 3 |
| 6 | 86-06-T_RS | 29 | 4 |
| 7 | IOP_123_1 | 10 | 5 |
| 8 | IOP_143_1 | 30 | 6 |
| 9 | IOP_250_1 | 12 | 6 |
| 10 | IOP_377_1 | 50 | 7 |
| 11 | IOP_411_1 | 43 | 8 |
| 12 | IOP_412_1 | 25 | 2 |
| 13 | IOP_439-G00 | 10 | 1 |
| 14 | IOP_450_1 | 35 | 3 |
| 15 | IOP_493_1 | 37 | 12 |
| 16 | IOP_494T-A2 | 26 | 7 |
| 17 | IOP_529_1 | 36 | 12 |
| 18 | IOP_557 | 35 | 5 |
| 19 | IOP_564_1 | 34 | 11 |
| 20 | IOP_579 | 25 | 3 |
| 21 | IOP_588-G00 | 20 | 4 |
| 22 | IOP_619_1 | 22 | 5 |
| 23 | IOP_644_1 | 35 | 6 |
| 24 | IOP_646_1 | 45 | 13 |
| 25 | IOP_64-G00 | 12 | 2 |
| 26 | IOP_Tumor_1 30 06az | 38 | 6 |
